# 3-(Ammonio­meth­yl)pyridinium dibromide

**DOI:** 10.1107/S1600536812040937

**Published:** 2012-10-03

**Authors:** Basem F. Ali, Rawhi Al-Far, Salim F. Haddad

**Affiliations:** a Department of Chemistry, Al al-Bayt University, Mafraq 25113, Jordan; b Faculty of Science and IT, Al-Balqa’a Applied University, Salt, Jordan; c Department of Chemistry, The University of Jordan, Amman 11942, Jordan

## Abstract

In the title salt, C_6_H_10_N_2_^2+^·2Br^−^, the non-H atoms of the 3-methyl­pyridinium unit of the cation are almost coplanar (r.m.s. deviation = 0.0052 Å). In the crystal, the dications and Br anions are linked by a combination of six hydrogen bonds, *viz.* one N_py_—H⋯Br, two C—H⋯Br and three H_2_N–H⋯Br, into supra­molecular layers, parallel to the *bc* plane, composed of alternating *R^2^_4_(10)* and *R^2^_4_(8)* loops. Weak π–π inter­actions between cationic rings with centroid–centroid distances of 3.891 (2) Å are also observed.

## Related literature

For non-covalent inter­actions, see: Allen *et al.* (1997 ▶); Desiraju (1997 ▶); Dolling *et al.* (2001 ▶); Gould *et al.* (1985 ▶); Hunter (1994 ▶); Hunter & Sanders (1990 ▶); Panunto *et al.* (1987 ▶); Robinson *et al.* (2000 ▶); Singh & Thornton (1990 ▶). For standard bond lengths, see: Allen *et al.* (1987 ▶). For graph-set notation, see: Bernstein *et al.* (1995 ▶).
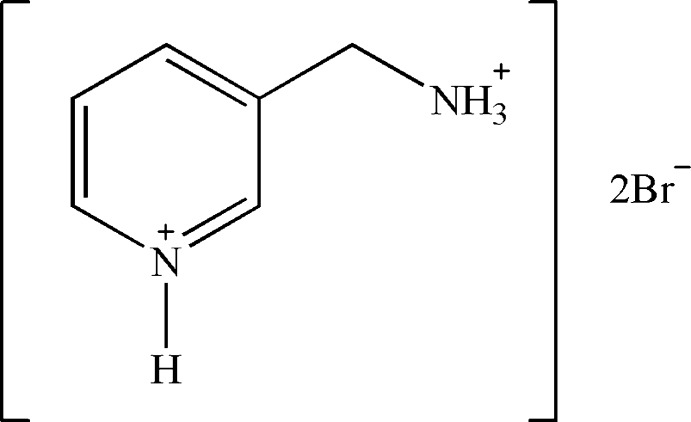


## Experimental

### 

#### Crystal data

C_6_H_10_N_2_^+^·2Br^−^
*M* *_r_* = 269.96
Monoclinic, 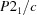

*a* = 11.1588 (6) Å
*b* = 9.3902 (5) Å
*c* = 9.3833 (5) Å
β = 113.092 (6)°
*V* = 904.44 (9) Å^3^
*Z* = 4
Mo *K*α radiation
μ = 8.90 mm^−1^
*T* = 293 K
0.23 × 0.18 × 0.12 mm

#### Data collection

Agilent Xcalibur EOS diffractometer
Absorption correction: multi-scan (*CrysAlis PRO*; Agilent, 2011 ▶) *T*_min_ = 0.143, *T*_max_ = 0.343
4032 measured reflections
2401 independent reflections
1641 reflections with *I* > 2σ(*I*)
*R*_int_ = 0.027

#### Refinement

*R*[*F* ^2^ > 2σ(*F* ^2^)] = 0.036
*wR*(*F* ^2^) = 0.070
*S* = 1.02
2401 reflections
92 parameters
H-atom parameters constrained
Δρ_max_ = 0.60 e Å^−3^
Δρ_min_ = −0.50 e Å^−3^

### 

Data collection: *CrysAlis PRO* (Agilent, 2011 ▶); cell refinement: *CrysAlis PRO*; data reduction: *CrysAlis PRO*; program(s) used to solve structure: *SHELXS97* (Sheldrick, 2008 ▶); program(s) used to refine structure: *SHELXL97* (Sheldrick, 2008 ▶); molecular graphics: *SHELXTL* (Sheldrick, 2008 ▶); software used to prepare material for publication: *SHELXTL*.

## Supplementary Material

Crystal structure: contains datablock(s) I, New_Global_Publ_Block. DOI: 10.1107/S1600536812040937/im2401sup1.cif

Structure factors: contains datablock(s) I. DOI: 10.1107/S1600536812040937/im2401Isup2.hkl

Supplementary material file. DOI: 10.1107/S1600536812040937/im2401Isup3.cml

Additional supplementary materials:  crystallographic information; 3D view; checkCIF report

## Figures and Tables

**Table 1:** Hydrogen-bond geometry (Å, °)

*D*—H⋯*A*	*D*—H	H⋯*A*	*D*⋯*A*	*D*—H⋯*A*
N2—H2*B*⋯Br1	0.89	2.61	3.358 (3)	142
N2—H2*C*⋯Br1^i^	0.89	2.69	3.330 (3)	130
N2—H2*D*⋯Br1^ii^	0.89	2.49	3.348 (3)	161
N1—H1*A*⋯Br2	0.86	2.41	3.206 (3)	155
C5—H5*A*⋯Br2^iii^	0.93	2.91	3.793 (4)	160
C6—H6*A*⋯Br2^iv^	0.93	2.89	3.619 (4)	136

Symmetry codes: (i) 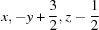; (ii) 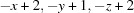; (iii) 
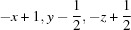
; (iv) 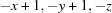.

**Fig. 1.**
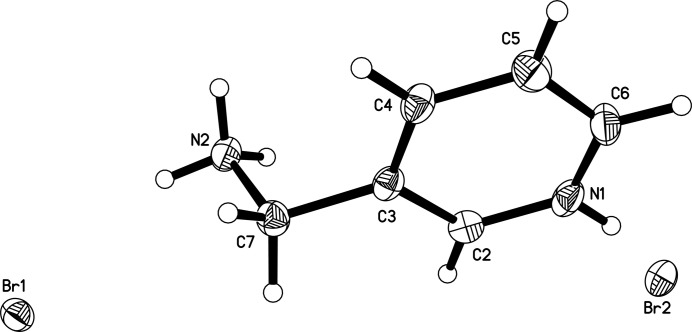
Molecular configuration of the 3-(ammoniomethyl)pyridinium cation and the bromide anions in the asymmetric unit. Displacement ellipsoids are drawn at the 30% probability level.

**Fig. 2.**
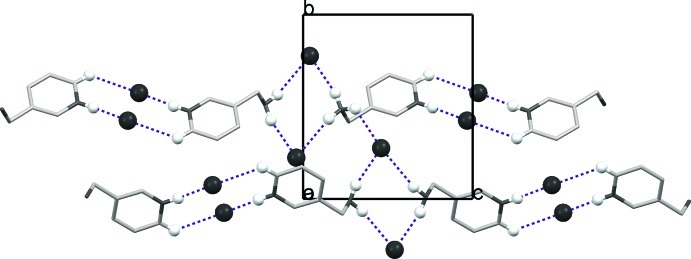
Cation···anion interactions assembled supramolecular layers parallel to bc plane. N—H···Br and C—H···Br hydrogen bonding interactions appears as dotted lines.

## supplementary crystallographic information

### Comment

Non-covalent interactions play an important role in organizing structural units in both natural and artificial systems (Desiraju, 1997). They exercise important effects on the organization and properties of many materials in areas such as biology (Hunter, 1994), crystal engineering (Allen *et al.*, 1997, Dolling *et al.*, 2001) and material science (Panunto *et al.*, 1987, Robinson *et al.*, 2000). We herein report the molecular structure of the salt, 3-(ammoniomethyl)pyridinium dibromide, along with it's supramolecular crystal structure.

In the title salt (Fig. 1) bond lengths (Allen *et al.*, 1987) and angles of the dication are within normal ranges. The unit (N1/C2/C3/C4/C5/C6/C7) of the independent cation is planar with r.m.s.d = 0.0052 (2) Å. The ammonium group largely deviates by 1.369 (7) Å out of this plane. The 3-methylammonium groups attached to the pyridinium ring through C3 shows a torsion angle of -87.9 (4)° for C2—C3—C7—N2.

The crystal packing involves extensive cation···anion interactions. These interactions assemble cations and anions into supramolecular layers parallel to the *bc* plane (Fig. 2) *via* N—H···Br and C—H···Br hydrogen bonding interactions of the types N_py_—H···Br, H_2_N—H···Br, and C—H···Br (Table 1). These layers are composed of alternating *R^2^_4_(10)* [two bromide anions and two (py)C/N—H units of two cations] and *R^2^_4_(8)* [two bromide anions and two ammonium groups *via* two H atoms each] graph set motifs (Bernstein *et al.*, 1995). Interlayer interactions are established through the third hydrogen of the ammonium group with a bromide anion of a next layer (Fig. 2).

The cations also interact to some extent by *offset face-to-face* interactions along the *a*-axis, adding extra lattice stability. This is evident by the centroid separation distances *C_1g_*···*C_1g_* (1 - *x*, 1 - *y*, 1 - *z*) of 3.891 (2) Å. The observed centroids separation distance is in accordance with those of calculated and the experimentally observed stacked (*offset-face-to-face*) interaction modes (Gould *et al.*, 1985, Hunter & Sanders, 1990, Hunter, 1994, Singh & Thornton, 1990). The N—H···Br and C—H···Br hydrogen bonding and aryl···aryl interactions consolidate to from a three-dimensional network.

### Experimental

The title compound was obtained unintentionally as the product of an attempted synthesis of a halo-stannate(II) organic-inorganic hybrids, using slow evaporation of an ethanolic hot mixture of solution of SnCl_2_.2H_2_O (1 mmol) and Br_2_(*l*) and solution of 3-methylaminopyridine (1 mmol) with 2 ml of HBr at room temperature. Crystals were grown from ethanol upon cooling and slow evaporation (yield: 78%). A suitable block shaped crystal cut from a larger colorless crystal was epoxy mounted on a glass fiber and the data collected at room temperature.

### Refinement

Hydrogen atoms were positioned geometrically, with N—H = 0.86 – 0.89 Å, C—H = 0.93 – 0.97 Å for aromatic H and C—H = 0.96 Å for methyl H, and constrained to ride on their parent atoms, *U*_iso_(H) = *xU*_eq_(*C,N*), where *x* = 1.5 for methyl H, and *x* = 1.2 for all other H atoms.

### Crystal data

**Table:**

C_6_H_10_N_2_^+^·2Br^−^	*F*(000) = 520
*M**_r_* = 269.96	*D*_x_ = 1.983 Mg m^−^^3^
Monoclinic, *P*2_1_/*c*	Mo *K*α radiation, λ = 0.71073 Å
Hall symbol: -P 2ybc	Cell parameters from 400 reflections
*a* = 11.1588 (6) Å	θ = 3.4–28.8°
*b* = 9.3902 (5) Å	µ = 8.90 mm^−^^1^
*c* = 9.3833 (5) Å	*T* = 293 K
β = 113.092 (6)°	Block, colorless
*V* = 904.44 (9) Å^3^	0.23 × 0.18 × 0.12 mm
*Z* = 4	

### Data collection

**Table:**

Agilent Xcalibur EOS diffractometer	2401 independent reflections
Radiation source: fine-focus sealed tube	1641 reflections with *I* > 2σ(*I*)
Graphite monochromator	*R*_int_ = 0.027
Detector resolution: 16.0534 pixels mm^-1^	θ_max_ = 29.0°, θ_min_ = 3.3°
ω scans	*h* = −14→15
Absorption correction: multi-scan (*CrysAlis PRO*; Agilent, 2011)	*k* = −6→12
*T*_min_ = 0.143, *T*_max_ = 0.343	*l* = −12→11
4032 measured reflections	

### Refinement

**Table:**

Refinement on *F*^2^	Primary atom site location: structure-invariant direct methods
Least-squares matrix: full	Secondary atom site location: difference Fourier map
*R*[*F*^2^ > 2σ(*F*^2^)] = 0.036	Hydrogen site location: inferred from neighbouring sites
*wR*(*F*^2^) = 0.070	H-atom parameters constrained
*S* = 1.02	*w* = 1/[σ^2^(*F*_o_^2^) + (0.0252*P*)^2^] where *P* = (*F*_o_^2^ + 2*F*_c_^2^)/3
2401 reflections	(Δ/σ)_max_ = 0.001
92 parameters	Δρ_max_ = 0.60 e Å^−^^3^
0 restraints	Δρ_min_ = −0.50 e Å^−^^3^

### Special details

**Table:**

Geometry. All e.s.d.'s (except the e.s.d. in the dihedral angle between two l.s. planes) are estimated using the full covariance matrix. The cell e.s.d.'s are taken into account individually in the estimation of e.s.d.'s in distances, angles and torsion angles; correlations between e.s.d.'s in cell parameters are only used when they are defined by crystal symmetry. An approximate (isotropic) treatment of cell e.s.d.'s is used for estimating e.s.d.'s involving l.s. planes.
Refinement. Refinement of *F*^2^ against ALL reflections. The weighted *R*-factor *wR* and goodness of fit *S* are based on *F*^2^, conventional *R*-factors *R* are based on *F*, with *F* set to zero for negative *F*^2^. The threshold expression of *F*^2^ > σ(*F*^2^) is used only for calculating *R*-factors(gt) *etc*. and is not relevant to the choice of reflections for refinement. *R*-factors based on *F*^2^ are statistically about twice as large as those based on *F*, and *R*- factors based on ALL data will be even larger.

### Fractional atomic coordinates and isotropic or equivalent isotropic displacement parameters (Å^2^)

**Table:**

	*x*	*y*	*z*	*U*_iso_*/*U*_eq_	
Br1	1.05938 (4)	0.77590 (4)	1.03996 (4)	0.03142 (12)	
N1	0.6363 (3)	0.4773 (4)	0.3288 (3)	0.0351 (8)	
H1A	0.6244	0.5040	0.2366	0.042*	
Br2	0.69216 (3)	0.57502 (5)	0.03324 (4)	0.03665 (14)	
N2	0.9797 (3)	0.5140 (3)	0.7751 (3)	0.0315 (8)	
H2B	1.0386	0.5659	0.8489	0.047*	
H2C	1.0002	0.5105	0.6926	0.047*	
H2D	0.9785	0.4262	0.8102	0.047*	
C2	0.7269 (3)	0.5432 (4)	0.4486 (4)	0.0295 (9)	
H2A	0.7761	0.6162	0.4315	0.035*	
C3	0.7476 (3)	0.5032 (4)	0.5971 (4)	0.0271 (9)	
C4	0.6744 (3)	0.3937 (4)	0.6182 (4)	0.0325 (9)	
H4A	0.6875	0.3647	0.7180	0.039*	
C5	0.5817 (4)	0.3262 (5)	0.4923 (4)	0.0399 (10)	
H5A	0.5326	0.2515	0.5062	0.048*	
C6	0.5634 (4)	0.3716 (5)	0.3462 (4)	0.0392 (10)	
H6A	0.5005	0.3288	0.2597	0.047*	
C7	0.8494 (3)	0.5797 (4)	0.7313 (4)	0.0321 (9)	
H7A	0.8533	0.6786	0.7036	0.039*	
H7B	0.8247	0.5774	0.8196	0.039*	

### Atomic displacement parameters (Å^2^)

**Table:**

	*U*^11^	*U*^22^	*U*^33^	*U*^12^	*U*^13^	*U*^23^
Br1	0.0375 (2)	0.0275 (2)	0.0308 (2)	−0.00378 (18)	0.01508 (16)	−0.00248 (17)
N1	0.0401 (19)	0.042 (2)	0.0205 (15)	0.0105 (18)	0.0088 (14)	0.0037 (15)
Br2	0.0324 (2)	0.0465 (3)	0.0321 (2)	0.0023 (2)	0.01373 (16)	0.00552 (19)
N2	0.0290 (16)	0.035 (2)	0.0273 (15)	−0.0019 (16)	0.0080 (13)	0.0035 (15)
C2	0.0294 (19)	0.031 (2)	0.033 (2)	0.0020 (18)	0.0170 (16)	0.0029 (18)
C3	0.0222 (18)	0.033 (2)	0.0257 (18)	0.0061 (18)	0.0095 (15)	0.0018 (17)
C4	0.0285 (19)	0.041 (3)	0.0271 (19)	−0.0018 (19)	0.0098 (15)	0.0064 (19)
C5	0.033 (2)	0.041 (3)	0.044 (2)	−0.009 (2)	0.0128 (18)	0.003 (2)
C6	0.032 (2)	0.043 (3)	0.033 (2)	−0.001 (2)	0.0021 (17)	−0.007 (2)
C7	0.031 (2)	0.034 (3)	0.0305 (19)	0.0019 (19)	0.0120 (16)	−0.0033 (18)

### Geometric parameters (Å, º)

**Table:**

N1—C6	1.333 (5)	C3—C4	1.375 (5)
N1—C2	1.333 (4)	C3—C7	1.507 (5)
N1—H1A	0.8600	C4—C5	1.382 (5)
N2—C7	1.482 (4)	C4—H4A	0.9300
N2—H2B	0.8900	C5—C6	1.373 (5)
N2—H2C	0.8900	C5—H5A	0.9300
N2—H2D	0.8900	C6—H6A	0.9300
C2—C3	1.372 (5)	C7—H7A	0.9700
C2—H2A	0.9300	C7—H7B	0.9700
			
C6—N1—C2	122.7 (3)	C3—C4—C5	120.5 (3)
C6—N1—H1A	118.6	C3—C4—H4A	119.8
C2—N1—H1A	118.6	C5—C4—H4A	119.8
C7—N2—H2B	109.5	C6—C5—C4	118.7 (4)
C7—N2—H2C	109.5	C6—C5—H5A	120.7
H2B—N2—H2C	109.5	C4—C5—H5A	120.7
C7—N2—H2D	109.5	N1—C6—C5	119.6 (4)
H2B—N2—H2D	109.5	N1—C6—H6A	120.2
H2C—N2—H2D	109.5	C5—C6—H6A	120.2
N1—C2—C3	119.9 (4)	N2—C7—C3	111.7 (3)
N1—C2—H2A	120.1	N2—C7—H7A	109.3
C3—C2—H2A	120.1	C3—C7—H7A	109.3
C2—C3—C4	118.6 (3)	N2—C7—H7B	109.3
C2—C3—C7	119.3 (3)	C3—C7—H7B	109.3
C4—C3—C7	122.1 (3)	H7A—C7—H7B	107.9
			
C6—N1—C2—C3	−0.3 (6)	C3—C4—C5—C6	−0.6 (6)
N1—C2—C3—C4	0.9 (5)	C2—N1—C6—C5	−0.6 (6)
N1—C2—C3—C7	−179.4 (3)	C4—C5—C6—N1	1.1 (6)
C2—C3—C4—C5	−0.4 (6)	C2—C3—C7—N2	−87.9 (4)
C7—C3—C4—C5	179.8 (4)	C4—C3—C7—N2	91.9 (4)

### Hydrogen-bond geometry (Å, º)

**Table:**

*D*—H···*A*	*D*—H	H···*A*	*D*···*A*	*D*—H···*A*
N2—H2*B*···Br1	0.89	2.61	3.358 (3)	142
N2—H2*C*···Br1^i^	0.89	2.69	3.330 (3)	130
N2—H2*D*···Br1^ii^	0.89	2.49	3.348 (3)	161
N1—H1*A*···Br2	0.86	2.41	3.206 (3)	155
C5—H5*A*···Br2^iii^	0.93	2.91	3.793 (4)	160
C6—H6*A*···Br2^iv^	0.93	2.89	3.619 (4)	136

Symmetry codes: (i) *x*, −*y*+3/2, *z*−1/2; (ii) −*x*+2, −*y*+1, −*z*+2; (iii) −*x*+1, *y*−1/2, −*z*+1/2; (iv) −*x*+1, −*y*+1, −*z*.
